# Preventing Peterson’s space hernia using a BIO synthetic mesh

**DOI:** 10.1186/s12893-021-01197-0

**Published:** 2021-05-04

**Authors:** Adam Skidmore, Edo O. Aarts

**Affiliations:** 1 Department of General Surgery, Victorian Obesity Surgery Centre, Localized in Warringal Hospital and Knox Private Hospital, 5 Burgundy Street, Heidelberg, Melbourne, VIC 3084 Australia; 2 WeightWorks Clinics, Surgical weight Loss Clinic, Amersfoort, The Netherlands

**Keywords:** Roux-en-Y gastric bypass, RYGB, Internal hernia, Hernia, Glue, BIO synthetic mesh, Mesh

## Abstract

**Background:**

Internal hernias occur after Roux-en-Y gastric bypass surgery (RYGB) when small bowel herniates into the intermesenteric spaces that have been created. The closure technique used is related to the internal hernia risks outcomes. Using a non-resorbable double layered suture, this risk can be significantly reduced from 8.9 to 2.5% in the first three postoperative years. By closing over a BIO mesh, the risk might be reduced even more.

**Setting:**

Two large private hospitals specialized in bariatric surgery.

**Methods:**

All patients receiving a RYGB for (morbid) obesity between 2014 and 2018 were included in this retrospective study. In all patients, the entero-enterostomy (EE) was closed using a double layered non-absorbable suture. In 2014, Peterson’s space was closed exclusively using glue, the years hereafter in a similar fashion as the EE, combined with a piece of glued BIO Mesh.

**Results:**

The glued RYGB patients showed 25% of patients with an internal hernia (14%) or open Peterson’s space compared to 0.5% of patients (p < 0.001) who had a combined sutured and BIO Mesh Closure of their Peterson’s space defect. Although this was an ideal technique for Peterson’s space, it led to 1% of entero-enterostomy kinking due to the firm adhesion formation.

**Conclusion:**

Gluing the intermesenteric spaces is not beneficial but placing a BIO Mesh in Peterson’s space is a promising new technique to induce local adhesions. It is above all safe, effective and led to an almost complete reduction of Peterson’s internal herniations. In the future, a randomized controlled trial comparing this technique to a double layered, non-absorbable suture should give more insights into which is the optimal closure technique.

## Background

The RYGB has been used for over five decades to battle morbid obesity [1] and continues to be widely performed. Besides a low perioperative risk [2], the efficacy of RYGB in the management of conditions such as Type 2 Diabetes continues to make RYGB the first choice for many surgeons and patients. Although RYGB has obvious benefits of the, it is no longer the most performed procedure. There are many reasons why this shift from RYGB to the Gastric Sleeve occurred worldwide [3]. One of these is that the RYGB comes with a risk of chronic or intermitting abdominal complaints.

These complaints have multiple origins, which range from obstipation to bacterial overgrowth. It seems however, that internal hernias (IH) are perhaps the biggest contributor. An IH can occur at any time after RYGB but the highest risk is when the maximum of weight loss has occurred. Normally this is between the first and the second year. IHs continue to be a problem after Laparoscopic Ante Colic RYGB (ACRYGB). Laparoscopic ACRYGB is associated with a higher incidence of internal hernia than open surgery [4, 5]. The lack of adhesions that are formed during laparoscopic surgery is thought to be the reason for this [6]. However, the increased efficiency, low rate of wound infections and for example incisional hernias makes a laparoscopic approach superior to an open one.

IHs occur when small bowel herniates into the intermesenteric spaces created when the roux limb is mobilised for anastomosis to the gastric pouch. In ACRYGB they can occur between the entero-enterostomy (EE) anastomosis or between the alimentary limb and the transverse mesocolon (Petersen’s Space). The incidence of symptomatic IHs  is quoted between 0.6 and 11% [7]. It has even been diagnosed as much as 16% in large retrospective studies [8–12].

To prevent these IHs occurring, many surgeons currently performing the procedure, close the mesenteric defects during primary RYGB. Using a non-resorbable double layered suture, this risk can be significantly reduced from 8.9 to 2.5% in the first three postoperative years [13]. However, the technique of closure seems to be related to these reduced IH risks outcomes, while for example, closure with staples, does reduce the number of internal hernias, but to a much lesser extent [14–16].

The techniques described in literature all use a mechanical method, but why not induce local adhesions that have proven (in open surgery) to lead to less IHs? This study reports on a novel technique and compares between the closure of Petersons space using glue alone vs Suture closure with a 3′0 Novafil v lock, reinforced with Bio A mesh (GORE® BIO-A®) to induce local adhesions.

## Methods

Patient who underwent a ACRYGB at the Warringal or Knox Private Hospital (Melbourne Australia), between January 2014 and July 2018 were included for this study. This timeframe was chosen to ensure a minimum of one year follow up after RYGB surgery and all procedures have been performed laparoscopically since 2014. Patients who did not meet at least one year of follow up were excluded. Ethics approval and consent to participate was obtained for this study at the ethics committee of the Warringal Hospital and Knox private hospital, Melbourne Australia. All procedures were performed by one experienced bariatric surgeon (> 1000 cases). Average time for surgery was 73 min. Average number of procedures performed per day was Two. All prospectively collected data were logged into a computerized research database starting from January 2014. In case of missing information, a detailed audit of all patients undergoing ACRYGB was also retrospectively reviewed.

During reoperation, a standardized step by step three port approach was used. The defects were always re-examined if the patient underwent further laparoscopy for other reasons or for IHs. This information was also prospectively recorded in the database.

### Approach to abdominal complaints

Although a wide range of abdominal complaints may occur, in most cases physicians commonly think it originates from the RYGB. This is especially the case once regular explanations are not found, such as infectious diseases. In our institution, blood withdrawal is routinely performed when acute abdominal complaints occur, including infectious parameters. When complaints like abdominal pain become sustained or recur (after 25 ug of fentanyl) or when they do not resolve in several hours, patients undergo a CT scan. When a diagnosis is found the patient is treated accordingly, but when no explanation is found there was a very low threshold for diagnostic laparoscopy.

### Definition internal hernia

An IH is defined as a bowel protruding through one (or more) of the defects or presence of chylous fluid with symptoms consistent with an internal hernia. A positive CT scan with mesenteric swirl or confirmed small bowel loops within an open Peterson or EE space are also considered to be diagnostic of an internal hernia when during laparoscopy there was at least one defect present.

### ACRYGB without BIO Mesh closure

All patients were operated on, using a standardized operative technique by the same experienced surgeon. A laparoscopic antecolic antegastric RYGB procedure was performed. First Treitz was identified and one meter of small intestine was measured which forms the biliary limb and stapled using a white cartridge (Echelon, Ethicon, Johnson & Johnson, New Brunswick, NJ, USA). Another meter of small intestine was measured from that point onwards, which forms the alimentary limb. The entero-enterotomy was performed with a 60-mm white linear Endo stapler combined with running absorbable suture (V-loc, Medtronic, Minneapolis, MN, USA). The EE defect was then closed with a continuous Novafil V lock suture (Medtronic). The alimentary limb was than sutured to the stomach in preparation of the gastro-jejunostomy. Petersons space was initially not closed but later closed as described below. A small amount of Glubran was also used. A long gastric pouch of 40–50 ml was constructed using a linear stapler along a 40Ch gastric tube. The gastro-jejunostomy was performed with a 60-mm white linear stapler, but only using 30 mm of length (ETS, Ethicon, Johnson & Johnson, New Brunswick, NJ, USA) combined with running barbed absorbable suture to close the anterior stapling gap (V-loc, Medtronic, Minneapolis, MN, USA). The integrity of the gastro-jejunostomy and gastric pouch staple line were tested intraoperatively for anastomotic leak with a burst test.

### ACRYGB with BIO mesh closure

**Fig. 1:**
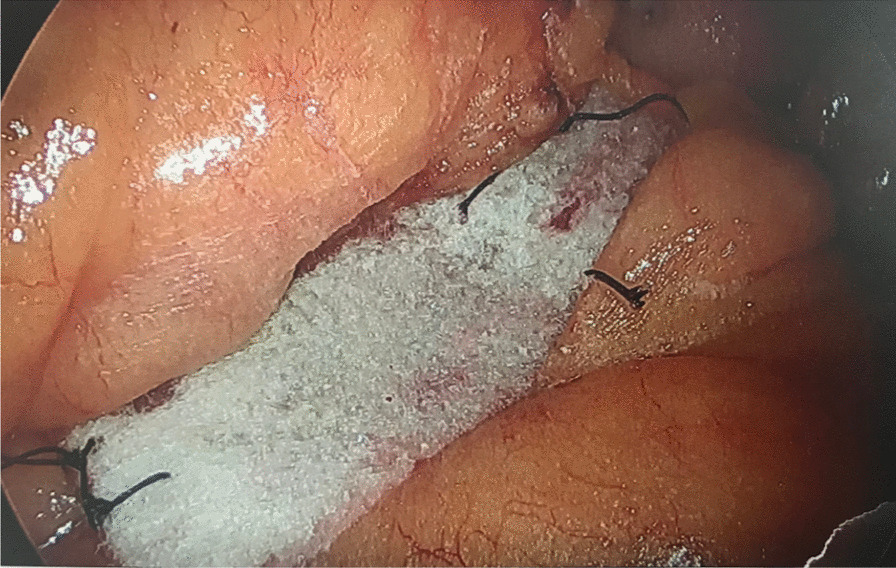
View of Peterson’s space after closure with BIO-A mesh with interrupted vicryl sutures

The whole procedure was performed in completely the same manner, but the closure technique of Peterson’s space and entero-enterostomy was changed. Closing of Peterson’s space and the entero-enterostomy were performed using a 3*1 and 4*2 cm piece of Bio A (GORE® BIO-A®, Newark Delaware USA) mesh ($580) respectively, placed over the sutured (V-loc, Medtronic, Minneapolis, MN, USA) closure and secured with a small amount of Glubran Glue (N-butyl-2-cyanoacrylate (NBCA). (Figs. 1, F2). Average time to close the space and place and secure mesh was 6 min.

**Fig. 2:**
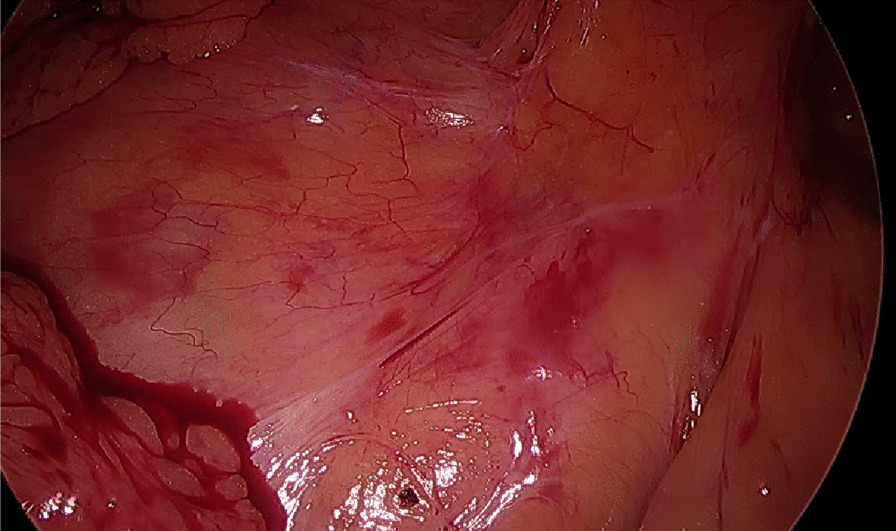
View of Peterson’s space two years after closure with BIO-A mesh

### Approach diagnostic laparoscopy

After introduction of the three ports, in most cases a liver retractor appeared unnecessary. First the pouch and gastro-enterotomy was examined and then the alimentary limb was measured, and length noted. Next, Peterson’s space and the entero-enterotomy were examined after Treitz was located, then, the common channel was measured. When too much traction occurred on the small bowel, the ileocolic angle was looked up and from there the common channel was followed up till the entero-enterotomy. With this approach all internal hernias could be solved.

### Statistical analysis

Data were analyzed using IBM® SPSS® (version 22.0 for Windows). Results are presented as mean values ± standard deviation (SD), unless specified otherwise. Descriptive statistics were used for demographic variables. Differences between groups were analyzed by Student’s *t* tests for continue variables and Fisher’s exact tests for categorical data. To adjust for the baseline covariates, i.e., age, sex, preoperative BMI, and preoperative diabetes, a linear regression analysis was performed. All tests were two tailed and a *p* value < 0.05 was considered as statistically significant.

## Results

All 297 RYGB cases performed in January 2014–July 2018 in the Warringal and Knox Private Hospitals were included. The first 75 patients underwent gluing of Peterson’s space and closure of the entero-enterostomy with a non-absorbable suture but no BIO mesh reinforcement. The remaining 170 patients had closure of the entero-enterostomy with suture and mesh and closure of Petersen’s space and the entero-enterostomy space with glue, suture and BIO mesh. Excluded were 19 and 33 patients respectively, because follow up > 12 months was missing. There were no significant differences between these groups in the baseline characteristics and the amount of weight loss at one and two years (Table 1). A good %Total Body Weight Loss (%TBWL) of 25.4% and 24.4% (p < 0.001 compared to preoperative, p = 0.09 between groups) for the Glue and BIO Mesh group patients respectively was achieved.

**Table 1:** Baseline and internal hernia characteristics

	2014 Glue	2015–2018 BIO mesh	p
Number of patients	75	170	
Female patients (%)	85%	88%	NS
Age	48 (17–69)	48 (18–72)	NS
BMI	44.9 (28.7–69.1)	43.7 (29.3–74.7)	NS
% Redo RYGB	89%	82%	NS
Mean follow up	36 months	22 months	** < 0.001**
%TBWL last visit	25.4%	24.4%	NS
∆BMI at herniation diagnosis	16.2	12.4 (n = 1)	NA

Bold indicate significant

*BMI* body mass index (kg/m^2^), *EE* entero-enterostomy, *%TBWL*  percent total body weight loss, *NS*  non significant, *NA*  not applicable

### Reoperations and internal herniation

Table 2 shows the indications for reoperations and their outcomes. No mortality occurred. 39 In the Glue and 52 Patients in the mesh group underwent further surgery and mandatory inspection of Petersen’s space was performed. In the Glue group there were eleven active hernias and eight open IH versus one open in the suture and Bio Mesh group. All these hernias in the Glue group were through Petersen’s space. Of the 52 patients who underwent a re-operation in the BIO Mesh group, all but one, had completely closed Petersen’s spaces and all had closed entero-enterostomy spaces (p < 0.001).

**Table 2:** Main outcomes for reoperations

	2014 Glue	2015–2017 BIO mesh	p
No reoperation	36 (48%)	120 (76%)	**0.019**
Reoperated patients	39 (52%)	50 (24%)	
Lap cholecystectomy	10 (13%)	19 (11%)	NS
Petersen’s hernia (%)	14 (25%)	1 (.5%)	** < 0.001**
EE Hernia (%)	0	0	NA
EE-kinking	0	2 (1%)	NS
Incisional hernia repair	5 (7%)	6 (4%)	NS
Hiatus hernia repair	3 (4%)	8 (5%)	NS
Diagnostic/adhesiolysis	2 (2%)	7 (4%)	NS
Volvulus	2 (2%)	1 (.5%)	NS
Right Hemicolectomy	1 (1%)	1 (.5%)	NS
Minimizer	8 (11%)	19 (11%)	NS

Bold indicate significant

*EE*  entero-enterostomy, *NS*  non significant, *NA*  not applicable

### Complications of closure

There were only two complications directly related to the closure of the mesenteric defects. These were however both related to the entero-enterostomy defect (mesh) and not the Petersen’s space. In both patients, there was kinking of the alimentary limb resulting in a proximal SBO. Both could be resolved during a diagnostic laparoscopy by diversion of the adhesions. Since January 2018 we stopped using the BIO Mesh for the entero-enterostomy and for this reason, only used it for Peterson’s space.

## Discussion

The exact incidence of IH after RYGB is hard to quantify. For example, Madan et al. reported no IH without closure of defects in 387 consecutive patients [17], while others report the incidence to be as high as 8.8% [18]. It is possible that the number of hernias that present with clinical significance is under-called for the unique clinical presentation which may be confused with other more common conditions. The lack of long-term follow-up and limited series make achieving statistical significance difficult. An important thing to remember is that although it is not a unique condition to Bariatric surgery, it is not well understood by the average emergency physician and General surgeon. With an increasing number of bypass type procedures being performed, this may become an increasing problem.

Additionally, there are twelve possible configurations of IHs which makes it challenging to diagnose them [19]. Patients often present with pain but little in the way of signs. Petersons Space hernias often present with significant symptoms and sometimes lead to severe complications like necrotising small bowel and even death when not acted upon swiftly enough. Often these patients have presented themselves to their primary care givers and emergency departments multiple times before a diagnosis is made. With cases of abdominal complaints or suspicion of internal herniation, many clinics perform CT scans to be better informed on a patient’s internal organs. CT scanning can, for example, show a mesenteric Swirl, which has a sensitivity of up to 90% [20] however this is not present in most patients with an active IH, and the condition can often be misdiagnosed as a variety of other conditions. Up to 20% of patients have no classic CT findings [12]. A CT scan without any findings should never be considered a reason not to perform a diagnostic laparoscopy. There remains consensus that there should be a low threshold for (laparoscopic) surgical exploration of these patients as long as diagnostic scanning will not improve their sensitivity by using, for example, 3D scanning [19]. In our clinic, as of January 2014, when starting/performing ACRYGB we protocolized that all patients with intermittent or persisting abdominal complaints should always undergo a diagnostic laparoscopy.

Although it can be argued that most bariatric surgeons would routinely close the defects [5] and that a number of studies would support routine closure [7, 11]. Some surgeons would argue reasons for non-closure [12], reasons such as mesenteric haematoma, kinking of bowel and problems with incomplete closure have been argued. It can also be argued that even with routine closure of the defects with absorbable or non-absorbable, suture can result in a failure rate of up to 83% [20]. The argument for non-closure also include the re-opening of these spaces with weight loss and the risk of incomplete closure leading to more risk of incarceration and strangulation. Some argue that rapid weight loss predisposes to development of internal hernia even with closure [21, 22]. Stenberg et al. however, already showed the effect of closure with non-absorbable sutures in a large RCT. Although an initial increase in reoperations because of entero-enterostomy kinking, a significant reduction was seen in the year’s hereafter [2]. Since this important RCT has been published, hardly anyone will argue the importance of defect closure.

Techniques for preventing IHs include leaving the jejunal mesentery intact [17], closing all spaces and using an ante colic technique even being advocated. Others have recommended using non-absorbable suture over absorbable suture [11]. There have been other case reports of using absorbable mesh as a plug within the space [17]. Scott et al. have described the use of absorbable mesh being stapled to the transverse mesocolon with considerable success [23].

In this study we compared gluing of Petersen’s space vs Closure with Non absorbable continuous suture, reinforced with a piece of BIO A Mesh. The purpose of the mesh reinforcement was to prevent the enlarging of the mesenteric defect resulting from fat melting after massive weight loss. The mesh would integrate and provide a framework for rigid scar formation over the repair, similar to an onlay hernia repair seen in incisional hernia repair or inguinal hernia repair. It would also prevent the issue related to incomplete closure allowing the mesh to potentially cover any areas that may not have been closed adequately. Choosing a BIO Mesh is not to have the theoretical complications of having a non-absorbable mesh on Treitz.

The technique proposed, is easy to perform and as of yet, has not been associated with any morbidity or complications. The only issue so far in 1% of patients is in relation to the entero enterostomy mesh placement with two Bowel obstructions related to the alimentary limb kinking on the mesh. There may, therefore, be an argument to not augment the entero-enterostomy closure and just close with non-absorbable sutures. It does, however, add to direct surgical costs due to the costs of the BIO Mesh and the (on average) 5 min added to the operation time. A factor that we must bear in mind, is that while it reduces the number of reoperations in these patients it seems beneficial for both patients and surgeons in the mid-long term.

Although data was obtained prospectively, this study is mainly observational. In our eyes a solid and thorough attempt has been made to compare two types of closure and this seems to result in favourable outcomes for the BIO Mesh group. These groups are in terms of baseline criteria comparable, but one must keep in mind that the mean period post RYGB of the glued group is a mean year longer. However, in this series the average mean follow up of 26 months is well over the mean time to internal hernia (11 months). Patients are instructed to come in for three visits (3, 6 and 12 months) in the first year and then annually thereafter. Due to the distances involved, patients do however, tend to come in only when complaints occur and mean follow up was therefore lowered. Additionally, a randomized controlled trial comparing two techniques as performed by Stenberg et al. is superior to this cohort setup. It would have been the ultimate study setup to perform a diagnostic laparoscopy in all patients after two years, but this is of course unethical.

## Conclusion

Internal herniation through Peterson’s space is one of the most common complications after RYGB. Closing this defect with clips or sutures partially reduces the chances on herniation, but not completely. Gluing this defect is not beneficial but placing a BIO Mesh in Peterson’s space is a promising new technique to induce local adhesions. It is at least safe, effective and has led to a complete reduction of Peterson’s internal herniations. In the future, a randomized controlled trial comparing this technique to a double layered, non-absorbable suture would give more insights in which is the optimal closure technique.

## Data Availability

The data supporting our findings are available in this article. The raw data sheets are not publicly available. Articles referred to can be found in the reference list.

## Abbreviations

ACRYGB: Ante Colic Roux-en-Y gastric bypass
CT: CAT scan
IH: Internal hernia
RCT: Randomised controlled trial
RYGB: Roux-en-Y gastric bypass
SBO: Small bowel obstruction
