# Correction to: Preventing Peterson’s space hernia using a BIO synthetic mesh

**DOI:** 10.1186/s12893-021-01295-z

**Published:** 2021-06-28

**Authors:** Adam Skidmore, Edo O. Aarts

**Affiliations:** 1 Department of General Surgery, Victorian Obesity Surgery Centre, Localized in Warringal Hospital and Knox Private Hospital, 5 Burgundy Street, Heidelberg, Melbourne, VIC 3084 Australia; 2 WeightWorks Clinics, Surgical Weight Loss Clinic, Amersfoort, The Netherlands

## Correction to: BMC Surg (2021) 21:236 https://doi.org/10.1186/s12893-021-01197-0

Following the publication of the original article [1], we were notified that “Petersen’s space” was wrongly spelled as “Peterson’s space”.

The original article has been corrected.
